# B Chromosomes – A Matter of Chromosome Drive

**DOI:** 10.3389/fpls.2017.00210

**Published:** 2017-02-15

**Authors:** Andreas Houben

**Affiliations:** Leibniz Institute of Plant Genetics and Crop Plant ResearchGatersleben, Germany

**Keywords:** supernumerary B chromosome, selfish element, non-disjunction, pollen mitosis, asymmetric cell division, pericentromere

## Abstract

B chromosomes are supernumerary chromosomes which are often preferentially inherited, deviating from usual Mendelian segregation. The balance between the so-called chromosome drive and the negative effects that the presence of Bs applies on the fitness of their host determines the frequency of Bs in a particular population. Drive is the key for understanding most B chromosomes. Drive occurs in many ways at pre-meiotic, meiotic or post-meiotic divisions, but the molecular mechanism remains unclear. The cellular mechanism of drive is reviewed based on the findings obtained for the B chromosomes of rye, maize and other species. How novel analytical tools will expand our ability to uncover the biology of B chromosome drive is discussed.

## Introduction

When transmission rates of chromosomes are higher than 0.5, not obeying the Mendelian law of equal segregation, the resulting transmission advantage is collectively referred to as ‘drive.’ Although B chromosomes (Bs) possibly show the most common form of drive known for genetic elements (Jones, 1991), little knowledge exists about the cellular and molecular mechanism behind their drive. Bs are not necessary for the growth and normal development of organisms, yet they are found in all eukaryotic phyla and are thought to stand for a specific type of selfish DNA (Kimura and Kayano, 1961; Jones and Rees, 1982; Jones, 1991; Burt and Trivers, 2006). Bs may vary in structure and chromatin properties in a species-specific way. Generally, it is assumed that Bs are derived from standard chromosomes (also called A chromosomes), either from the same or from a related species (reviewed in Camacho et al., 2000; Jones and Houben, 2003; Houben et al., 2014; Valente et al., 2016). Beside B chromosomes, various other genetic elements promote their own transmission at the expense of other components of the genome. Best studied examples of drive that correspond to autosomal distorters are the *t* haplotype in mouse, *Spore killer* in fungi and *Segregation Distorter* (*SD*) in *Drosophila* (Lyttle, 1991). Naturally occurring sex chromosome-linked meiotic drive with impact on the sex-ratio has been reported mainly in Rodentia and Diptera (reviewed in Helleu et al., 2015).

Drive of B chromosomes occur at pre-meiotic, meiotic or post-meiotic divisions in a species-specific way. Beside drive, the non-Mendelian inheritance of Bs could also be effected by mitotic and meiotic instability. The maximum number of Bs tolerated by the host varies between species (e.g., maize, chives and rye could carry up to 34, 20, 6 Bs, respectively) and depends on a balance between B chromosomes accumulation based on drive, and B chromosome caused negative effects, especially on fertility and vigor [effects induced by Bs are listed in Bougourd and Jones ()]. However, not all B carrying species possess a drive mechanism. In these species, it is likely that counteracting advantageous features have to be in action to maintain the B polymorphism [examples listed in Jones (1991)]. While previous reviews on Bs already gave comprehensive overviews on the evolution and general significance of the B chromosome drive (Jones, 1991; Burt and Trivers, 2006), this review will focus on the potential mechanism behind the drive resulting in a higher than expected number of Bs in the next generation.

## The Mechanism of B Chromosome Drive in rye and Other Species

**FIGURE 1:**
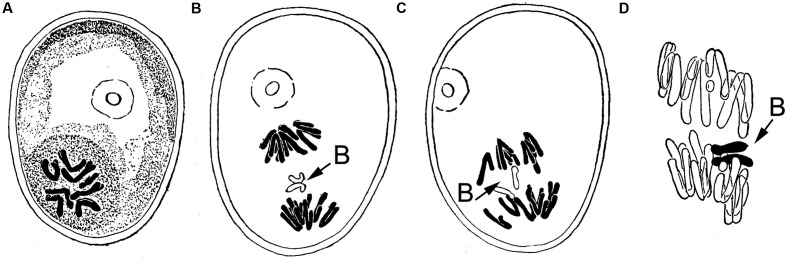
**Hand drawing of Hasegawa (1934) depicting for the first time the process of B chromosome drive at first pollen mitosis in rye (A)** metaphase, **(B)** lagging B chromosome due to non-disjunction. **(C)** Disjoined sister chromatids of the B chromosome going to different poles, **(D)** chromosome drive occurs, the future generative nucleus receives both sister chromatids of the B, in contrast the vegetative nucleus contains only the 7 standard A chromosomes. Permission has been obtained for use of copyrighted material from the Japan Mendel Society.

The post-meiotic drive of the rye (*Secale cereale* L.) B chromosome is one of the best analyzed mechanisms amongst Bs. Hasegawa (1934) noted first the unusual behavior of B chromosomes during the first pollen grain mitosis in rye. He described the B behavior as following, ‘……the two split halves (sister chromatids) of the extra chromosomes are in most cases included in the generative nucleus in late anaphase.’ His observations were summarized in marvelous hand drawings shown in **Figure 1**. He observed that during anaphase of the first pollen mitosis the two B chromatids do not split and in most cases both B chromatids became part of the generative nucleus. Based on this observation he concluded ‘…from the irregular distribution of the extra (B) chromosome, the plants having 14, 15, and 16 chromosomes in diploid may be expected in the offspring of 8-chromosome rye’ (note, at this time the term B chromosome did not yet exist and a rye plant possessing 2Bs was called ‘8-chromosome rye,’ while the normal rye has seven pairs of chromosomes). The frequency of non-disjunction at first pollen mitosis depends on the genotype (Puertas et al., 1998, 2000). At second pollen mitosis B sister chromatids divide normally like standard chromosomes. In rye drive has been found in plants with up to six Bs (Kishikawa, 1965). In crosses 0B × 2B or 2B × 0B, plants with up to 4Bs are obtained in the progenies. However, plants with odd numbers of Bs were only rarely observed (Müntzing, 1945).Based on this observation; it was assumed that a similar drive occurs in female gametophytes. Indeed, Håkanson (1948) observed anaphase cells with lagging Bs also in the embryo-sac during first post-meiotic division. A similar drive of Bs during first pollen mitosis was found in the *Triticeae* species. *Aegilops mutica* and *A. speltoides.* (Mendelson and Zohary, 1972; Ohta, 1996). Notably, B non-disjunction works as well when the supernumerary chromosome of rye is introduced as an extra chromosome into *S. vavilovii* (Puertas et al., 1985), hexaploid wheat (Lindström, 1965; Müntzing, 1970; Niwa et al., 1997; Endo et al., 2008) or hypo-pentaploid *Triticale* (Kishikawa and Suzuki, 1982). Hence, the B chromosome regulates the process of non-disjunction on its own (Matthews and Jones, 1983; Romera et al., 1991).

**FIGURE 2:**
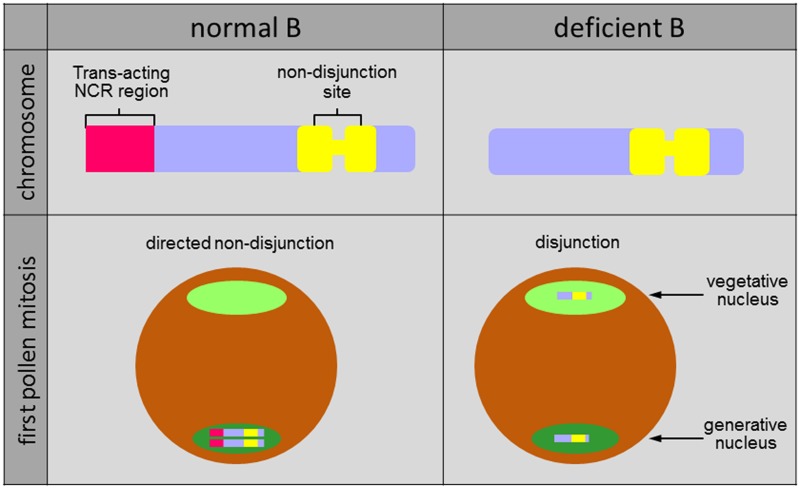
**Structure and drive of rye B chromosomes**. The *trans*-acting non-disjunction control region (NCR) is enriched in B-specific satellite DNAs expressing non-coding RNA. During first pollen mitosis Bs undergo non-disjunction and both chromatids are included preferentially in the generative nucleus. In contrast, due to the absence of the NCR deficient Bs segregate during the first pollen mitosis like standard A chromosomes.

Analysis of B chromosome variants allowed the identification of the region controlling the process of non-disjunction at the end of the long chromosome arm. Rye Bs lacking the so-called non-disjunction control region (NCR) (e.g., iso-short arm Bs) undergo normal disjunction at first pollen anaphase [(Müntzing, 1945, 1948; Håkanson, 1959; Endo et al., 2008), **Figure 2**]. The NCR can act *in trans* because non-disjunction works for the standard and the deficient B chromosome, if a standard B or the NCR-containing region of the long B arm is present in the same cell (e.g., translocated to an A chromosome) processing a deficient B (Lima-de-Faria, 1962; Endo et al., 2008). In the heterochromatic NCR several B-specific satellite DNAs reside (Sandery et al., 1990; Blunden et al., 1993; Carchilan et al., 2007; Klemme et al., 2013). The NCR is also labeled with the euchromatin-specific posttranslational histone mark H3K4me3 (Carchilan et al., 2007). The observation that some NCR-specific satellites produce long-non-coding RNA predominantly in anthers (Carchilan et al., 2007) could indicate the possible involvement of NCR-derived non-coding RNA in maintaining cohesion in key regions of B-sister chromatids, preventing proper segregation. Likely the failure in mitotic segregation reflects a malfunction to correctly resolve the pericentromeric heterochromatin of the B chromosome during first pollen mitosis in rye.

Albeit no similarity between NCR- and B (peri)centromere-located sequences has been described, it is of interest that some likeness exists at the protein level between a part of the NCR-located satellite repeat E3900 (which encodes a partial *gag* protein of a Ty3/gypsy-type LTR retrotransposon) and the conserved centromeric repeats osrch3 and CentC of rice and maize, respectively (Langdon et al., 2000). On the other hand, sequence similarity between non-coding RNA and the target region is not required. For example, the regions of the Xist (X-inactive specific transcript) non-coding RNA that are necessary for the localization on the inactivated sex X chromosome have no noticeable similarity at the sequence level (Wutz et al., 2002). Hence, it is possible that B chromosome encoded non-coding RNAs block access to necessary factors at specific genomic loci such as the B pericentromere; alternatively the B-derived non-coding RNAs could act as “guide molecules” to direct protein complexes (Banaei-Moghaddam et al., 2012).

Alternatively or in addition the recent identification of a surprisingly high number of B-encoded transcripts in a number of species, e.g., in rye (Martis et al., 2012; Banaei-Moghaddam et al., 2013; Ma et al., 2016), fish (Silva et al., 2014; Valente et al., 2014; Ramos et al., 2016), *Drosophila* (Bauerly et al., 2014) and cervids (Makunin et al., 2016), provides the basis to hypothesize about the involvement of protein-coding genes, or pseudogenes in non-disjunction control. For example, a B-specific non-disjunction control gene might exist in analogy to the mechanism of sex chromosome drive in *Drosophila*. In *Drosophila simulans* the rapidly evolving X-linked heterochromatin protein 1 gene, *HP1D2*, has an important function in the Paris-type sex ratio meiotic drive system (Helleu et al., 2016).

## The Composition of the (Peri)Centromere Differs Between A and B Chromosomes

Comparison between rye A and B centromeres revealed differences in the (peri)centromere repeat composition (Banaei-Moghaddam et al., 2012). The B centromere shares the same known repeats as the centromere of As. But in addition, the B pericentromere is extended with the B-specific repeat ScCl11 and mitochondrion-derived DNA. Both sequences do not interact with CENH3-containing nucleosomes (Banaei-Moghaddam et al., 2012). One might imagine that the centromere of the B evolved from a standard centromere and additonal repeats accumulated in the centromere of the newly formed B chromosome. A comparable distinct composition was also observed for the centromere of the maize B chromosome. CentC repeats and centromere-specific retrotransposons of the maize B are disrupted by species-specific B-centromeric sequences (Jin et al., 2005).

The accumulation of B-specific repeats in the pericentromere takes probably part in the organization of pericentric heterochromatin, which as we know plays a role in chromosome segregation (Yamagishi et al., 2008). Heterochromatin is essential for proper sister chromatid cohesion, e.g., in *Schizosaccharomyces pombe*, repeats next to the kinetochore are required for proper sister chromatid cohesion (Bernard et al., 2001).

## Cellular Events During B Chromosome Drive in Rye and Other Species

In a number of plant species the microtubule spindle is asymmetric during the first pollen grain mitosis. The asymmetry of this division plays a critical role in the subsequent formation of the unequal daughter cells, the generative and the vegetative one (Twell, 2010). Due to the asymmetric division and the formation of two nuclei having different degrees of chromatin condensation, in rye the A chromosome centromeres are clustered in the condensed generative nucleus, whereas in the less condensed vegetative nucleus the centromeres are scattered over a larger area (Banaei-Moghaddam et al., 2012). Notably, in the generative nucleus the centromeres A and standard B chromosomes do not intermingle. In contrast, Bs lacking the NCR-region, cluster together with the A chromosomes. Thus, the distinct interphase position of standard Bs in the generative nucleus is likely due to their lagging behind the separated A chromatids during anaphase of the first pollen mitosis (Banaei-Moghaddam et al., 2012).

Taken the asymmetric geometry of the spindle at first pollen mitosis in consideration it is likely as by Jones (1991) suggested, that the inclusion of Bs in the generative nucleus is caused by the fact that the equatorial plate is nearer to the generative pole and lagging Bs are passively included in the generative nucleus. Alternatively, due to a higher pulling force on the B centromere toward the generative pole Bs may preferentially accumulate in the generative nucleus (Banaei-Moghaddam et al., 2012). Asymmetrical spindles are likely also key component of the meiotic drive of the B chromosomes in the grasshopper *Myrmeleotettix maculatus* (Hewitt, 1976) and the premeiotic drive of Bs in the Asteraceae *Crepis capillaris* (Rutishauser and Rothlisberger, 1966). Thus, asymmetry of the microtubule spindle seems to be a component of the B accumulation mechanisms.

In maize, the drive of Bs requires a factor located on the long arm of the B that may act *in trans.* The maize B drive mechanism involves non-disjunction at the second pollen grain mitosis, placing two copies of the B into one of the two sperm. The sperm carrying Bs preferential fertilize the egg (Roman, 1947; Carlson, 1978; Lamb et al., 2006). Characterizing an epigenetically silenced maize B centromere demonstrated that non-disjunction does not depend on a functional centromere (Han et al., 2007). In maize, non-disjunction of Bs also takes place in endosperm and tapetum cells (Alfenito and Birchler, 1990; Chiavarino et al., 2000). In tapetum cells, Bs mediate instability of A chromosomes (Gonzalez-Sanchez et al., 2004). Sporophytic non-disjunction of the B occurs mainly if this supernumerary chromosome is present at high copy number, implying that non-disjunction is repressed if the number of Bs is low (Masonbrink and Birchler, 2010). One factor encoded by a maize standard A chromosome seems to influence the B accumulation process (Gonzalez-Sanchez et al., 2003). Sperm nuclei carrying deletion derivatives of the translocation chromosome B-9 (involving parts of the standard A chromosome 9 and the B), which lack the centric heterochromatin and possibly some euchromatin of the B, no longer have the capacity for preferential fertilization of the egg (Carlson, 2007). Thus, although the B chromosomes of rye and maize originated independently (Martis et al., 2012), similar drive mechanisms in both cereals evolved in parallel.

## Future Perspectives

Considering that Bs would not exist without a drive it is about time to decipher this process at the molecular level. With the development of novel analytical tools a better understanding of this intriguing mechanism becomes possible. Sufficient genome sequence information is available for some of the B chromosome carrying species or could be generated to search for candidate genes of non-coding transcripts involved in the process of drive. Comparative transcript analysis of genotypes with a different degree of B non-disjunction could be used to identify non-disjunction-linked transcripts. Genome editing methods will become instrumental to analyze the function of sequences involved in non-disjunction. Emerging techniques of chromatin imaging (e.g., CRISPR-FISH) allow the labeling of defined genomic regions in living cells, useful to decipher the spatio-temporal distribution of Bs; and even to witness the process of chromosome drive in living cells. The combination of innovative technologies will expand our ability to uncover the mystery of B chromosome drive.

## Author Contributions

The author confirms being the sole contributor of this work and approved it for publication.

## Conflict of Interest Statement

The author declares that the research was conducted in the absence of any commercial or financial relationships that could be construed as a potential conflict of interest.

## References

[B1] Alfenito M. Birchler J. (1990). Studies on B chromosome stability during development. *Maydica* 35 359–366.

[B2] Banaei-Moghaddam A. M. Meier K. Karimi-Ashtiyani R. Houben A. (2013). Formation and expression of pseudogenes on the B chromosome of rye. *Plant Cell* 25 2536–2544. 10.1105/tpc.113.111856 23839789 PMC3753381

[B3] Banaei-Moghaddam A. M. Schubert V. Kumke K. Weiβ O. Klemme S. Nagaki K. (2012). Nondisjunction in favor of a chromosome: the mechanism of rye B chromosome drive during pollen mitosis. *Plant Cell* 24 4124–4134. 10.1105/tpc.112.105270 23104833 PMC3517240

[B4] Bauerly E. Hughes S. E. Vietti D. R. Miller D. E. McDowell W. Hawley R. S. (2014). Discovery of supernumerary B chromosomes in *Drosophila melanogaster*. *Genetics* 196 1007–1016. 10.1534/genetics.113.160556 24478336 PMC4286233

[B5] Bernard P. Maure J. F. Partridge J. F. Genier S. Javerzat J. P. Allshire R. C. (2001). Requirement of heterochromatin for cohesion at centromeres. *Science* 294 2539–2542. 10.1126/science.1064027 11598266

[B6] Blunden R. Wilkes T. J. Forster J. W. Jimenez M. M. Sandery M. J. Karp A. (1993). Identification of the E3900 family, a 2nd family of rye chromosome-B specific repeated sequences. *Genome* 36 706–711. 10.1139/g93-095 18470018

[B7] Bougourd S. M. Jones R. N. (1997). B chromosomes: a physiological enigma. *New Phytol.* 137 43–54. 10.1046/j.1469-8137.1997.00823.x

[B8] Burt A. Trivers R. (2006). *Genes in Conflict: The Biology of Selfish Genetic Elements*. Cambridge, MA: The Belknap Press of Harvard University Press, 602 10.4159/9780674029118

[B9] Camacho J. P. M. Sharbel T. F. Beukeboom L. W. (2000). B-chromosome evolution. *Philos. Trans. R. Soc. B Biol. Sci.* 355 163–178. 10.1098/rstb.2000.0556 PMC1692730 10724453

[B10] Carchilan M. Delgado M. Ribeiro T. Costa-Nunes P. Caperta A. Morais-Cecílio L. (2007). Transcriptionally active heterochromatin in rye B chromosomes. *Plant Cell* 19 1738–1749. 10.1105/tpc.106.046946 17586652 PMC1955731

[B11] Carlson W. R. (1978). B-chromosome of corn. *Annu. Rev. Genet.* 12 5–23. 10.1146/annurev.ge.12.120178.000253 371533

[B12] Carlson W. R. (2007). Locating a site on the maize B chromosome that controls preferential fertilization. *Genome* 50 578–587. 10.1139/G07-035 17632579

[B13] Chiavarino A. M. Rosato M. Manzanero S. Jiménez G. González-Sánchez M. Puertas M. J. (2000). Chromosome nondisjunction and instabilities in tapetal cells are affected by B chromosomes in maize. *Genetics* 155 889–897. 10835407 10.1093/genetics/155.2.889 PMC1461132

[B14] Endo T. R. Nasuda S. Jones N. Dou Q. Akahori A. Wakimoto M. (2008). Dissection of rye B chromosomes, and nondisjunction properties of the dissected segments in a common wheat background. *Genes Genet. Syst.* 83 23–30. 10.1266/ggs.83.23 18379131

[B15] Gonzalez-Sanchez M. Gonzalez-Gonzalez E. Molina F. Chiavarino A. M. Rosato M. Puertas M. J. (2003). One gene determines maize B chromosome accumulation by preferential fertilisation; another gene(s) determines their meiotic loss. *Heredity* 90 122–129. 10.1038/sj.hdy.6800185 12634817

[B16] Gonzalez-Sanchez M. Rosato M. Chiavarino M. Puertas M. J. (2004). Chromosome instabilities and programmed cell death in tapetal cells of maize with B chromosomes and effects on pollen viability. *Genetics* 166 999–1009. 10.1534/genetics.166.2.999 15020483 PMC1470749

[B17] Håkanson A. (1948). Behaviour of accessory rye chromosomes in the embryo sac. *Hereditas* 34 35–59. 10.1111/j.1601-5223.1948.tb02826.x

[B18] Håkanson A. (1959). Behaviour of different small accessry rye chromosomes at pollen mitosis. *Hereditas* 45 623–631. 10.1111/j.1601-5223.1959.tb03071.x

[B19] Han F. Lamb J. C. Yu W. Gao Z. Birchler J. A. (2007). Centromere function and nondisjunction are independent components of the maize B chromosome accumulation mechanism. *Plant Cell* 19 524–533. 10.1105/tpc.106.049577 17322406 PMC1867328

[B20] Hasegawa N. (1934). A cytological study on 8-chromosome rye. *Cytologia* 6 68–77. 10.1508/cytologia.6.68

[B21] Helleu Q. Gerard P. R. Dubruille R. Ogereau D. Prud’homme B. Loppin B. (2016). Rapid evolution of a Y-chromosome heterochromatin protein underlies sex chromosome meiotic drive. *Proc. Natl. Acad. Sci. U.S.A.* 113 4110–4115. 10.1073/pnas.1519332113 26979956 PMC4839453

[B22] Helleu Q. Gerard P. R. Montchamp-Moreau C. (2015). Sex chromosome drive. *Cold Spring Harb. Perspect. Biol.* 7 a017616. 10.1101/cshperspect.a017616 PMC4315933 25524548

[B23] Hewitt G. M. (1976). Meiotic drive for B-chromosomes in the primary oocytes of *Myrmeleotettix maculatus* (Orthopera: Acrididae). *Chromosoma* 56 381–391. 10.1007/BF00292957 949923

[B24] Houben A. Banaei-Moghaddam A. M. Klemme S. Timmis J. N. (2014). Evolution and biology of supernumerary B chromosomes. *Cell. Mol. Life Sci.* 71 467–478. 10.1007/s00018-013-1437-7 23912901 PMC11113615

[B25] Jin W. Lamb J. C. Vega J. M. Dawe R. K. Birchler J. A. Jiang J. (2005). Molecular and functional dissection of the maize B chromosome centromere. *Plant Cell* 17 1412–1423. 10.1105/tpc.104.030643 15805482 PMC1091764

[B26] Jones N. Houben A. (2003). B chromosomes in plants: escapees from the A chromosome genome? *Trends Plant Sci.* 8 417–423. 10.1016/S1360-1385(03)00187-0 13678908

[B27] Jones R. N. (1991). B-chromosome drive. *Am. Nat.* 137 430–442. 10.1086/285175

[B28] Jones R. N. Rees H. (1982). *B Chromosomes*, 1st Edn. New York, NY: Academic Press.

[B29] Kimura M. Kayano H. (1961). The maintenance of supernumerary chromosomes in wild populations of *Lillium callosum* by preferential segregation. *Genetics* 46 1699–1712. 14456042 10.1093/genetics/46.12.1699 PMC1210178

[B30] Kishikawa H. (1965). Cytogenetic studies of B chromosomes in rye, *Secale cereale* L in Japan. *Agric. Bull. Saga Univ.* 21 1–81.

[B31] Kishikawa H. Suzuki A. (1982). Cytological study on hypo-pentaploid Triticale with four B chromosomes of rye. *Jpn. J. Genet.* 57 17–24. 10.1266/jjg.57.17

[B32] Klemme S. Banaei-Moghaddam A. M. Macas J. Wicker T. Novák P. Houben A. (2013). High-copy sequences reveal distinct evolution of the rye B chromosome. *New Phytol.* 199 550–558. 10.1111/nph.12289 23614816

[B33] Lamb J. C. Han F. Auger D. L. Birchler J. A. (2006). A trans-acting factor required for non-disjunction of the B chromosome is located distal to the TB-4Lb breakpoint on the B chromosome. *Maize Genet. Coop. News Lett.* 80 51–54.

[B34] Langdon T. Seago C. Jones R. N. Ougham H. Thomas H. Forster J. W. (2000). De novo evolution of satellite DNA on the rye B chromosome. *Genetics* 154 869–884. 10655237 10.1093/genetics/154.2.869 PMC1460944

[B35] Lima-de-Faria A. (1962). Genetic interaction in rye expressed at chromosome phenotype. *Genetics* 47 1455–1462. 17248134 10.1093/genetics/47.10.1455 PMC1210295

[B36] Lindström J. (1965). Transfer to wheat of accessory chromosomes from rye. *Hereditas* 54 149–155. 10.1111/j.1601-5223.1965.tb02012.x

[B37] Lyttle T. W. (1991). Segregation distorters. *Annu. Rev. Genet.* 25 511–557. 10.1146/annurev.ge.25.120191.002455 1812815

[B38] Ma W. Gabriel T. S. Martis M. M. Gursinsky T. Schubert V. Vrána J. (2016). Rye B chromosomes encode a functional Argonaute-like protein with in vitro slicer activities similar to its A chromosome paralog. *New Phytol.* 213 916–928. 10.1111/nph.14110 27468091

[B39] Makunin A. I. Kichigin I. G. Larkin D. M. O’Brien P. C. Ferguson-Smith M. A. Yang F. (2016). Contrasting origin of B chromosomes in two cervids (Siberian roe deer and grey brocket deer) unravelled by chromosome-specific DNA sequencing. *BMC Genomics* 17:618 10.1186/s12864-016-2933-6 PMC4982142 27516089

[B40] Martis M. M. Klemme S. Banaei-Moghaddam A. M. Blattner F. R. Macas J. Schmutzer T. (2012). Selfish supernumerary chromosome reveals its origin as a mosaic of host genome and organellar sequences. *Proc. Natl. Acad. Sci. U.S.A.* 109 13343–13346. 10.1073/pnas.1204237109 22847450 PMC3421217

[B41] Masonbrink R. E. Birchler J. A. (2010). Sporophytic nondisjunction of the maize B chromosome at high copy numbers. *J. Genet. Genomics* 37 79–84. 10.1016/S1673-8527(09)60027-8 20171580

[B42] Matthews R. B. Jones R. N. (1983). Dynamics of the B chromosome polymorphism in rye II. Estimates of parameters. *Heredity* 50 119–137. 10.1038/hdy.1983.14

[B43] Mendelson D. Zohary D. (1972). Behavior and transmission of supernumerary chromosomes in *Aegilops speltoides*. *Heredity* 29 329–339. 10.1038/hdy.1972.97

[B44] Müntzing A. (1945). Cytological studies of extra fragment chromosomes in rye. II. Transmission and multiplication of standard fragments and iso-fragments. *Hereditas* 31 457–477. 10.1111/j.1601-5223.1945.tb02763.x 21021079

[B45] Müntzing A. (1948). Cytological studies of extra fragment chromosomes in rye. V. A new fragment type arisen by deletion. *Hereditas* 34 435–442. 10.1111/j.1601-5223.1948.tb02853.x

[B46] Müntzing A. (1970). Chromosomal variation in the Lindström strain of wheat carrying accessory chromosomes in rye. *Hereditas* 66 279–286. 10.1111/j.1601-5223.1970.tb02351.x

[B47] Niwa K. Horiuchi G. Hirai Y. (1997). Production and characterization of common wheat with B chromosomes of rye from Korea. *Hereditas* 126 139–146. 10.1111/j.1601-5223.1997.00139.x

[B48] Ohta S. (1996). Mechanisms of B-chromosome accumulation in *Aegilops mutica* Boiss. *Genes Genet. Syst.* 71 23–29. 10.1266/ggs.71.23

[B49] Puertas M. Jimenez G. Manzanero S. Chiavarino A. M. Rosato M. Naranjo C. A. (2000). Genetic control of B chromosome transmission in maize and rye. *Chromosomes Today* 13 79–92. 10.1007/978-3-0348-8484-6_7

[B50] Puertas M. J. Jiménez G. Manzanero S. Chiavarino A. M. Rosato M. Naranjo C. A. (1998). Genetic control of B chromosome transmission in maize and rye. *Cytogenet. Cell Genet.* 81 103–103.

[B51] Puertas M. J. Romera F. Delapena A. (1985). Comparison of B-chromosome effects on *Secale cereale* and *Secale vavilovii*. *Heredity* 55 229–234. 10.1038/hdy.1985.95

[B52] Ramos E. Cardoso A. L. Brown J. Marques D. F. Fantinatti B. E. Cabral-de-Mello D. C. (2016). The repetitive DNA element BncDNA, enriched in the B chromosome of the cichlid fish *Astatotilapia* latifasciata, transcribes a potentially noncoding RNA. *Chromosoma* (in press). 10.1007/s00412-016-0601-x 27169573

[B53] Roman H. (1947). Mitotic nondisjunction in the case of interchanges involving the B-type chromosome in maize. *Genetics* 32 391–409. 17247252 10.1093/genetics/32.4.391 PMC1209386

[B54] Romera F. Jimenez M. M. Puertas M. J. (1991). Factors controlling the dynamics of the B-chromosome polymorphism in Korean rye. *Heredity* 67 189–195. 10.1038/hdy.1991.79

[B55] Rutishauser A. Rothlisberger E. (1966). Boosting mechanism of B-chromosomes in *Crepis* capilaris. *Chromosomes Today* 1 28–30.

[B56] Sandery M. J. Forster J. W. Blunden R. Jones R. N. (1990). Identification of a family of repeated sequences on the rye B-chromosome. *Genome* 33 908–913. 10.1139/g90-137 18470018

[B57] Silva D. M. Pansonato-Alves J. C. Utsunomia R. Araya-Jaime C. Ruiz-Ruano F. J. Daniel S. N. (2014). Delimiting the origin of a B chromosome by FISH mapping, chromosome painting and DNA sequence analysis in Astyanax paranae (Teleostei, Characiformes). *PLoS ONE* 9:e94896 10.1371/journal.pone.0094896 PMC3988084 24736529

[B58] Twell D. (2010). Male gametogenesis and germline specification in flowering plants. *Sex. Plant Reprod.* 24 149–160. 10.1007/s00497-010-0157-5 21103996

[B59] Valente G. T. Conte M. A. Fantinatti B. E. Cabral-de-Mello D. C. Carvalho R. F. Vicari M. R. (2014). Origin and evolution of B chromosomes in the Cichlid fish *Astatotilapia* latifasciata based on integrated genomic analyses. *Mol. Biol. Evol.* 31 2061–2072. 10.1093/molbev/msu148 24770715

[B60] Valente G. T. Nakajima R. T. Fantinatti B. E. Marques D. F. Almeida R. O. Simões R. P. (2016). B chromosomes: from cytogenetics to systems biology. *Chromosoma* [Epub ahead of print]. 10.1007/s00412-016-0613-6 27558128

[B61] Wutz A. Rasmussen T. P. Jaenisch R. (2002). Chromosomal silencing and localization are mediated by different domains of Xist RNA. *Nat. Genet.* 30 167–174. 10.1038/ng820 11780141

[B62] Yamagishi Y. Sakuno T. Shimura M. Watanabe Y. (2008). Heterochromatin links to centromeric protection by recruiting shugoshin. *Nature* 455 251–255. 10.1038/nature07217 18716626

